# Effects of sustained daily latanoprost application on anterior chamber anatomy and physiology in mice

**DOI:** 10.1038/s41598-018-31280-1

**Published:** 2018-08-30

**Authors:** Laura M. Dutca, Danielle Rudd, Victor Robles, Anat Galor, Mona K. Garvin, Michael G. Anderson

**Affiliations:** 1 Center for Prevention and Treatment of Visual Loss Iowa City Veterans Administration Medical Center, Iowa City, IA USA; 2 Department of Ophthalmology and Visual Science, University of Iowa, Iowa City, IA USA; 3 Electrical and Computer Engineering, University of Iowa, Iowa City, IA USA; 4 Miami Veterans Administration Medical Center and Bascom Palmer Institute, University of Miami, Miami, FL USA; 5 Molecular Physiology and Biophysics, University of Iowa, Iowa City, IA USA

## Abstract

Latanoprost is a common glaucoma medication. Here, we study longitudinal effects of sustained latanoprost treatment on intraocular pressure (IOP) in C57BL/6J mice, as well as two potential side-effects, changes in iris pigmentation and central corneal thickness (CCT). Male C57BL/6J mice were treated daily for 16 weeks with latanoprost. Control mice were treated on the same schedule with the preservative used with latanoprost, benzalkonium chloride (BAK), or handled, without ocular treatments. IOP and CCT were studied at pre-treatment, 2 “early” time points, and 2 “late” time points; slit-lamp analysis performed at a late time point; and expression of corneal and iridial candidate genes analyzed at the end of the experiment. Latanoprost lowered IOP short, but not long-term. Sustained application of BAK consistently resulted in significant corneal thinning, whereas sustained treatment with latanoprost resulted in smaller and less consistent changes. Neither treatment affected iris pigmentation, corneal matrix metalloprotease expression or iridial pigment-related genes expression. In summary, latanoprost initially lowered IOP in C57BL/6J mice, but became less effective with sustained treatment, likely due to physiological adaptation. These results identify a new resource for studying changes in responsiveness associated with long-term treatment with latanoprost and highlight detrimental effects of commonly used preservative BAK.

## Introduction

It is estimated that by 2020, 76 million people worldwide will be afflicted with glaucoma^1^. Although multiple factors contribute to risk for developing this disease, only one is currently therapeutically modifiable: elevated intraocular pressure (IOP). Thus, all current treatments for glaucoma focus on efforts to lower IOP. The medications most commonly used for this purpose are prostaglandin (PG) analogs such as latanoprost, a synthetic isopropyl ester analog of prostaglandin F_2α._ The mechanism(s) underlying this effect of PG activity remains unclear but is known to involve substantial increases in uveoscleral outflow^2^. Latanoprost is currently the most prescribed PG analog, and the recommended dosage is one drop in the affected eye, once daily in the evening^3^. Although it has a favorable safety/efficacy profile^2,4^, it has some shortcomings. Specifically, some patients fail to respond^5–7^, others become insensitive over time^8^, and some experience adverse side-effects^2,9–11^.

Animal models are crucial tools for both studying how drugs such as latanoprost lead to improved disease outcomes and, ultimately, improving their performance. The development of latanoprost for the reduction of IOP relied largely upon studies using monkeys and dogs^12–15^. Cats and rabbits were also tested, but both have non-specific or unusual responses to PGs^16,17^ and thus their utilization in IOP studies was limited. More recently latanoprost was found to reduce IOP in mice^18–20^, and since then they have been used to: study mechanisms whereby latanoprost influences IOP^21–24^, test the IOP-lowering abilities of latanoprost-related PG derivatives and analogs^25–28^, and study side effects of latanoprost^29,30^. Despite the many ways in which animal models have contributed to research involving latanoprost, and their wide-spread use in studies of glaucoma in general, one area that has not been well studied involves the consequences of long-term latanoprost use in models such as mice.

Here, we characterize the longitudinal effects of short- and long-term latanoprost treatment on IOP in the C57BL/6J mouse strain, using a protocol that mimics the treatment regimen for glaucoma patients (once daily administration for a long period of time). Among the many reported side-effects of long term latanoprost use that could be studied with mice, we limited our current study to two that have been frequent subjects of human studies (changes in iris pigmentation and changes in corneal thickness), which happen to also coincide with expertise of our group. At the conclusion of the longitudinal study, we also performed a gene expression analysis of the cornea and iris, focusing on candidate genes that are likely to be influenced by latanoprost.

## Methods

### Mice

All experiments were performed at the University of Iowa, conducted in accordance with the Association for Research in Vision and Ophthalmology Statement for the Use of Animals in Ophthalmic and Vision Research, and approved by the Animal Care and Use Committee of the University of Iowa. Male C57BL/6J mice, 8 weeks of age, were obtained from The Jackson Laboratory (Bar Harbor, ME, USA). Because C57BL/6J is an inbred mouse strain, all individuals are for practical purposes genetically identical, and thus highly similar, having irises of dark brown color^31^ and with minimal variation in CCT and IOP^32–34^. The mice were maintained in a cyclical pattern of 12 hours lights on followed by 12 hours of lights off, for the duration of the study. After 2 weeks of acclimatization in the facility, the mice were randomly split into 3 groups of 6: (1) Lat (experimental) group, both eyes to be treated with a latanoprost ophthalmic solution 0.005%, that also contains BAK 0.02% as a preservative (Bausch & Lomb Inc., Rochester, N.Y., USA) on a daily basis; (2) BAK (control 1) group, both eyes to be treated with 0.02% solution of the preservative BAK in 0.1 M phosphate buffer and 0.4% sodium chloride (pH 6.7) on a daily basis; and (3) Naïve (control 2) group, to be handled in identical fashion to the other groups without receiving any ocular treatment. During the study 2 mice died (1 from the BAK group and 1 from the Naïve group). No mice were otherwise excluded from the study and no efforts were made to identify or exclude individual non-responders. IOP and CCT phenotypes were studied pre-treatment (for both IOP and CCT at 11 weeks of age, week 0 of the experiment), at 2 “early” time points (for both IOP and CCT after 1 and 2 weeks of treatment), and 2 “late” time points (for CCT after 11 and 12 weeks of treatment; for IOP after 14 and 15 weeks of treatment). Slit-lamp analysis was performed after 14 weeks of treatment, and euthanasia was performed after 16 weeks of treatment. Slit-lamp exams, and photodocumentation, were performed by MGA, who has extensive experience with this kind of examination^31,35,36^. At the start of treatment, the mice were 11 weeks old. Once a day (between 08:30–09:30 am) for 16 weeks, the mice were treated with 5 μL of drug (Lat), were treated with 5 μL of preservative solution (BAK), or were handled without receiving ocular treatments (Naïve).

### Measurement of IOP

All IOP measurements were performed 2–4 hours after treatment (11:00–13:00), to minimize the effects of diurnal IOP variation. Beforehand, the mice were acclimatized (1 hour) to the room in which the measurements were to be performed. The IOP of both eyes of each mouse was measured using the TonoLab rebound tonometer (Icare Finland Oy, Helsinki, Finland). Mice were anesthetized with a mixture of 2.5% isoflurane and 100% oxygen, and the readings were performed during the first 5 minutes after the start of anesthesia. IOP values were determined based on an average of 5 independent readings gathered within 5 minutes of anesthesia, with an inclusion criteria that there be less than a 3 mmHg difference between the readings (there were two data collection attempts excluded, IOP of 1 eye from the 1-wk post-treatment BAK cohort and IOP of 1 eye from the 15-wk post-treatment Lat cohort). Averages were analyzed by ANOVA with Tukey’s multi-comparison post-hoc test, using GraphPad Prism version 7.00 for Windows (GraphPad Software, La Jolla, CA, USA). Based on the standard deviation of the mean IOP for each cohort at the pre-treatment age (Naïve 0.89 mmHg, BAK 1.06 mmHg, Lat 1.96 mmHg), the study had 80% power (α = 0.05) to detect differences of 1.17 mmHg, 1.4 mmHg, and 2.35 mmHg, respectively, for a two-sample *t*-test. The person providing daily eye drops to the mice was the same as the person performing IOP measurements and was thus familiar by sight with the individual mice and was not masked.

### Measurement of CCT

Mice were anesthetized with ketamine/xylazine (intraperitoneal injection of 100 mg ketamine and 10 mg xylazine/kg body weight; Ketaset, Fort Dodge Animal Health, Fort Dodge, IA, USA; AnaSed, Lloyd Laboratories, Shenandoah, IA, USA). A heating pad provided supplemental indirect warmth during both the induction of anesthesia and recovery. Immediately after anesthesia, the eyes were hydrated with balanced salt solution (BSS; Alcon Laboratories, Fort Worth, TX, USA). After recordings were completed, yohimbine was administered to avoid potential side effects of anesthesia^37^ (intraperitoneal injection 2 mg/kg of body weight; Yobine®, Lloyd, Inc., Shenandoah, IA, USA) and the mouse was placed into an empty cage for recovery. Corneal images of both eyes of each mouse were obtained using a Bioptigen SD-OCT (Bioptigen, Inc., Durham, NC, USA) with a 12-mm telecentric bore, a reference arm position of 1048, and with the pupil centered in the volume intensity projection. Scan parameters were as follows: radial volume scans 2.0 mm in diameter, 1000 A-scans/B-scan, 100 B-scans/volume, 1 frame/B-scan, 1 volume and each A-scan had 1024 pixels. For each eye, the CCT and the thicknesses of the epithelium and stroma were determined using vertical angle-locked B-scan calipers and the Bioptigen InVivoVue computer software. The inside edges of the calipers were aligned with the respective surfaces as described (see Supplemental Fig. S1)^38^. Results were analyzed using repeated measures paired ANOVA with Dunnett correction for multiple comparisons. Based on the standard deviation of the mean CCT for each cohort at the pre-treatment age (Naïve 3.24 μm, BAK 4.1 μm, Lat 2.68 μm), the study had 80% power (α = 0.05) to detect differences of 4.3 μm, 5.4 μm, and 3.2 μm, respectively, for a two-sample *t*-test. CCT data were collected in a masked fashion by a person separate from the one providing the daily eye drops to the mice.

### Automated segmentation of CCT images

A multi-resolution graph-based approach was also used, as described previously^38^, to segment the same images used to determine CCT manually as explained in the preceding section. Briefly, the volumes were preprocessed to remove any speckle noise, and then down-sampled by a factor of four and two. For all resolutions, volumetric surface-cost images (i.e., c1 (x, y, z), c2 (x, y, z), and c3 (x, y, z)) were derived from the volume as inputs to the graph-theoretic approach. Cost images were generated using a 3D recursive Gaussian filter. For each image slice (400 rows by 200 columns), 200 thicknesses were determined between the two surfaces, resulting in 20,000 measurements of thickness for the 100 slices per volume. These were averaged to obtain a mean CCT value.

### Slit-lamp examination

The slit-lamp examination was performed after 14 weeks of treatment with latanoprost. Anterior chamber phenotypes were assessed in conscious mice, using a slit lamp at 25 × magnification (SL-D7; Topcon, Tokyo, Japan), and photodocumented using a digital camera (D800; Nikon, Tokyo, Japan). All photographs were taken in a single session, with: identical slit-lamp settings; the slit-lamp arm in the same position; and identical camera settings. For the iris images shown in the figure, the images were cropped and reduced in size. For the quantification of iris color, digital images were analyzed using Adobe Photoshop software (Adobe Systems Inc., San Jose, CA, USA). For 1 in-focus image of the left eye of each mouse, RGB-values were measured for a sample area of each iris. The Elliptical Marquee tool was used to select sample areas (250 pixels × 250 pixels, approximately the size of the pupil) in the same location of each image, approximately one pupil width away from the pupil. Slight vertical adjustment was allowed to avoid photography artifacts. For each sample, the “blur-average” filter was applied, and then the Eyedropper tool was used to measure the R-, G-, and B-values of each sample area. These values were averaged for each treatment group and compared using ANOVA with Tukey’s multi-comparison post-hoc test.

### Tissue collection, RNA extraction, and RT-PCR

Immediately after the mice were euthanized, enucleated eyes were dissected in RNA*later* solution (Ambion, Applied Biosystems, Carlsbad, CA, USA) and the cornea and iris were collected. Both corneas, and separately both irises, from each mouse were pooled, and the samples were lysed and homogenized using a tissue grinder (Tissue-Tearor, Biospec Products, Inc) for 1.5 min on ice. Total RNA was extracted using the miRVana™ microRNA Isolation Kit (Ambion, Life Technologies, Carlsbad CA, USA), and traces of genomic DNA were removed using the DNA-*free* kit (Ambion, Applied Biosystems, Carlsbad, CA, USA) following the manufacturer’s instructions. RNA was reverse transcribed using the iScript™ cDNA Synthesis Kit (Bio-Rad Laboratories Inc., Hercules, CA, USA) according to the manufacturer’s instructions. Most of the primers for qPCR were designed using Primer-BLAST^39^; the exceptions were those for *β-actin* and *Mmp3*, which were designed using the Primer 3 software^40^. The sequences of all primers are provided in Supplemental Table S1. Quantitative PCR was performed using a SYBR green mastermix (iQ SYBR Green Supermix; Bio-Rad Laboratories, Hercules, CA, USA) and a real-time PCR detection system (C1000 Thermal Cycler; Bio-Rad Laboratories, Hercules, CA, USA). The composition of the reaction mix was 1x SYBR green mastermix, 200 nM of each primer and 1 ng/uL RNA. PCR conditions were: 95 °C for 3 minutes, followed by 40 × (95 °C for 10 seconds, 60 °C for 20 seconds). PCR products were subjected to melting-curve analysis to ensure that only a single product was amplified. Each experiment included 3 technical replicates of each RNA sample. The data were analyzed using Bio-Rad CFX Manager software. Primer efficiency was generally determined using a standard curve constructed with 5 points for each tissue. The exception was the *Mmp9* primer set, for which retinal tissue was used, because the efficiency with the corneal tissue was too low. For each transcript, *C*_t_ values for each sample were determined using Bio-Rad CFX Manager, and then averaged and normalized to values for *β-actin* and *HPRT* (hypoxanthine-guanine phosphoribosyltransferase). Statistical analysis was performed using ANOVA with Tukey’s multi-comparison post-hoc test.

## Results

### Treatment with latanoprost lowers IOP in the short, but not long term

**Figure 1:**
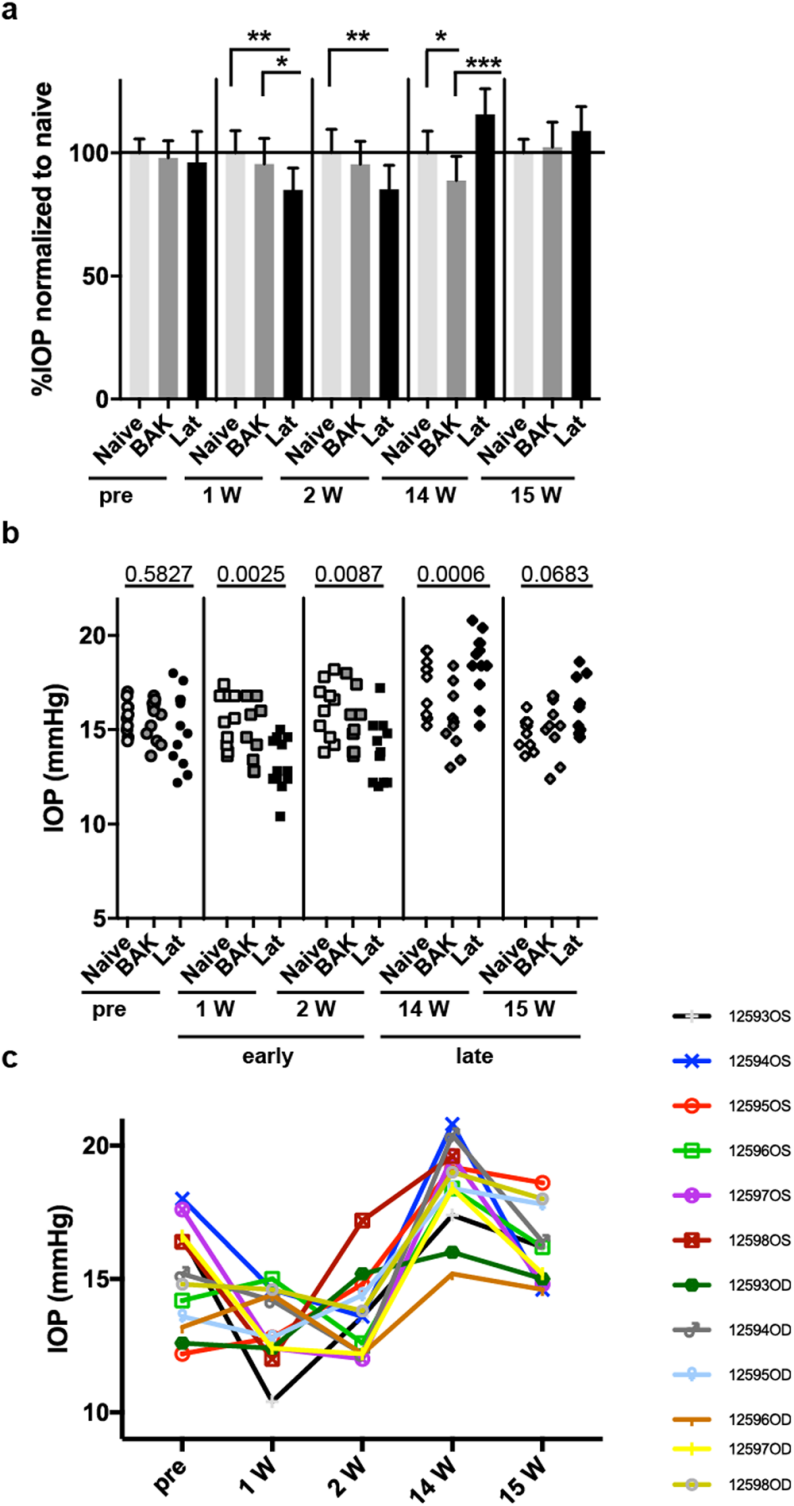
Effects of latanoprost and BAK treatments on IOP in C57BL/6J mice. (**a**) Mean (±SD) IOP values for the indicated treatment groups at pre, early (1 week and 2 weeks), and late (14 and 15 weeks) time points, normalized to the mean of the Naïve group at that time point. A One-way ANOVA with Tukey’s multi-comparison post-hoc test was performed and the results of the multiple comparisons are shown as: ^*^P < 0.05, ^**^P < 0.01, ^***^P < 0.001. (**b**) Scatter plot of all IOP data with the corresponding *P* value for the ANOVA analysis indicated. n = 5 mice and 10 data points (Naïve), 5 mice and 10 data points (BAK, note that week 1 had 9 data points) and 6 mice and 12 data points (Lat, note that week 15 had 11 data points). (**c**) Same data as in panel (b), but plotted as IOP measurements over time for each eye of the Lat group (see Supplemental Figure S2 for additional groups).

In order to study the effects of daily latanoprost application on mouse eyes, we obtained a cohort of C57BL/6J mice from a commercial source, randomly assigned individual mice to 1 of 3 groups (Lat, BAK, or Naïve) and, after 2 weeks of acclimatization to our facility, initiated our protocol. Prior to treatment (pre-treatment) the mean IOPs of the Lat, BAK, and Naïve groups were indistinguishable (15.08 ± 1.96 mmHg, 15.53 ± 1.06 mmHg, and 15.68 ± 0.89 mmHg, respectively). Thereafter, each morning, the mice were treated with 0.005% latanoprost (Lat), were treated with 0.02% benzalkonium chloride (BAK), or were handled (Naive), but did not receive ocular treatment. The IOP for each eye was measured and compared to that in eyes from mice in the Naïve and BAK treated groups, two weeks in a row at early (weeks 1 and 2), and two weeks in a row at late (weeks 14 and 15) time points. Early in the treatment period at week 1, the mean IOP for the Lat group (13.17 ± 1.38 mmHg) was significantly lower than that for either the Naïve (15.5 ± 1.40 mmHg; *p* = 0.0024) or BAK-treated (14.8 ± 1.612 mmHg; *p* = 0.0435) group (Fig. 1a,b). At week 2, the mean IOP for the Lat group (13.87 ± 1.59 mmHg) was still significantly lower than that for the Naïve group (16.02 ± 1.52 mmHg; *p* = 0.0076), but not for the BAK group (15.28 ± 1.49 mmHg; *p* = 0.0984). However, late in the treatment period Lat no longer lowered IOP. After 14 weeks of treatment, the IOP of the Lat group (18.53 ± 1.66 mmHg) was actually higher than that in both the Naïve (17.42 ± 1.53 mmHg; *p* = 0.26) and BAK-treated groups (15.46 ± 1.73 mmHg; *p* = 0.0004) (Fig. 1). After 15 weeks of treatment, latanoprost remained ineffective, with no statistically significant differences in IOP between the groups, but a continued trend for elevated IOP within the Lat group (16.13 ± 1.45 mmHg, Lat; 14.8 ± 0.83 mmHg, Naïve; 15.14 ± 1.51 mmHg, BAK) (Fig. 1b). Collectively, these results indicate that although latanoprost is initially effective in reducing IOP in C57BL/6 J mice, after several weeks of treatment it loses efficacy.

### Long-term treatment with BAK consistently leads to significant thinning of the cornea

**Figure 2:**
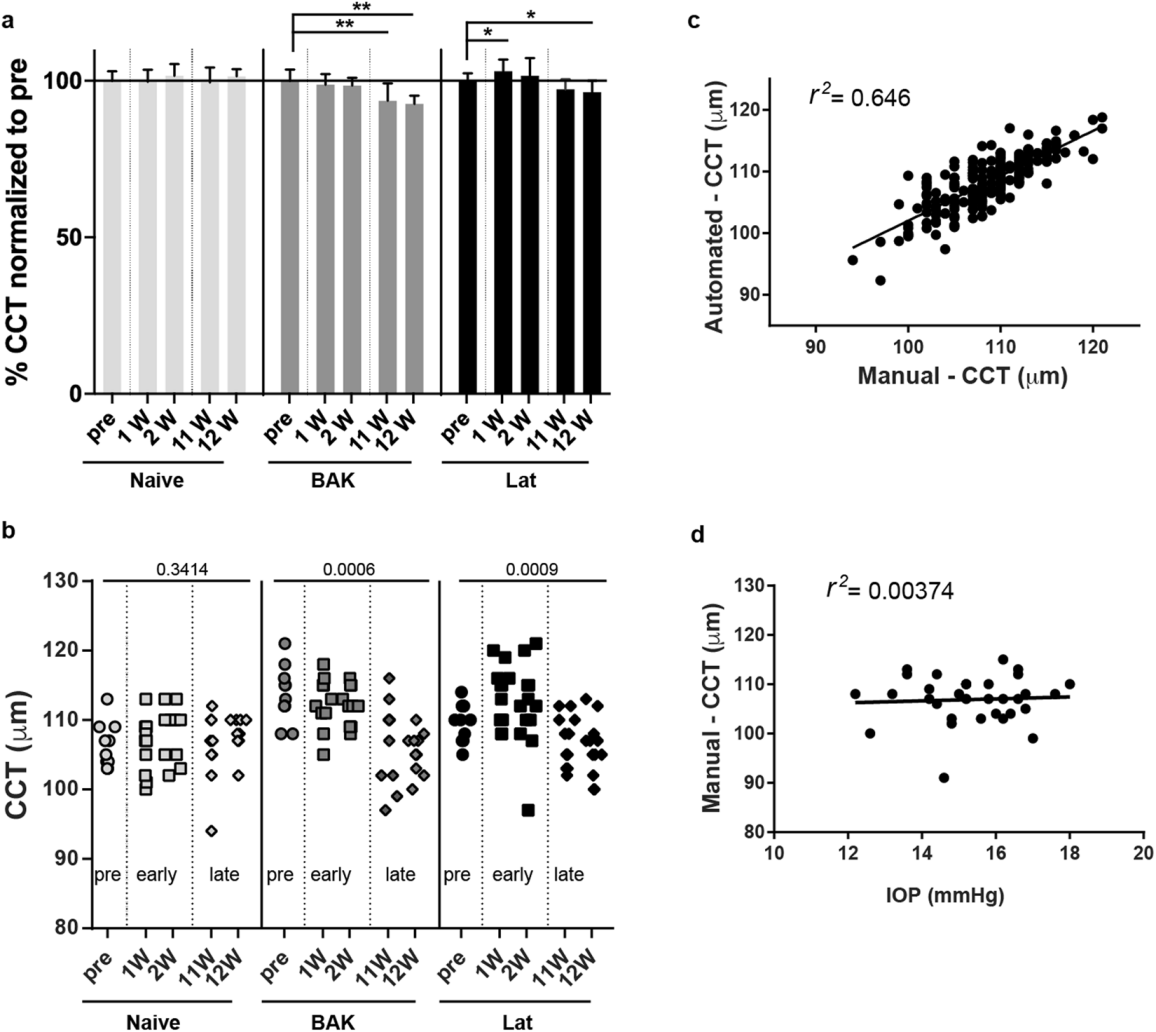
Effects of latanoprost and BAK treatments on the CCT in C57BL/6J mice. (**a**) Mean (±SD) CCT values pre, early (1 week and 2 weeks), and late (11 and 12 weeks) time points in Naive, BAK, and Lat treatments groups, normalized to the mean of the CCT determined pre-treatment. A repeated measures paired ANOVA analysis with the Dunnett post*-*hoc test was performed and the results of the multiple comparison are shown as: ^*^P < 0.05, ^**^P < 0.01. Also note, the pattern of changes in CCT for each cohort does not match the changes in IOP, see Fig. 1a. (**b**) Scatter plot of all CCT data with the corresponding *P* value for the repeated measures paired ANOVA analysis indicated. (**c**) Correlation of manually and automatically determined CCT values. (**d**) Correlation of manually determined CCT and IOP for all eyes at the pre-treatment time point. n = 5 mice (Naïve), 5 mice (BAK), and 6 mice (Lat).

Changes in CCT are a well-documented side-effect of latanoprost use in humans^41–43^. In order to determine whether sustained latanoprost treatment has the same effect in mice, CCT of the 3 groups was analyzed manually at baseline, early (weeks 1 and 2) and late (weeks 11 and 12) time points. In the case of the Naïve group total CCT did not change significantly across the pre-treatment, early, and late time points (106.4 ± 3.24 µm, 106.1 ± 4.09 µm, 108.1 ± 4.12 µm, 105.9 ± 5.09 µm, 107.9 ± 2.47 µm; respectively), indicating that total CCT does not change detectably as a function of age in C57BL/6 J mice (Fig. 2a,b). In the case of the BAK group, total CCT decreased over the course of the experiment (113.8 ± 4.10 µm, 112.4 ± 3.89 µm, 112.1 ± 2.77 µm, 106.6 ± 6.29 µm, 105.4 ± 3.03 µm for pre-treatment, weeks 1, 2, 11 and 12, respectively), with a significant difference between the late versus pre-treatment time points (*p* = 0.0046 and *p* = 0.0051). For the Lat group, total CCT changed in a more complex pattern (109.9 ± 2.68 µm, 113.3 ± 4.12 µm, 111.7 ± 6.30 µm, 106.9 ± 3.55 µm, 105.9 ± 4.08 for pre-treatment, weeks 1, 2, 11 and 12, respectively), with a significant increase in total CCT from the pre-treatment to week 1 (*p* = 0.0192) followed by a decrease from pre-treatment to week 12 (*p* = 0.0236). Because the Lat group was treated with a commercial formulation of latanoprost that also contains the same concentration of BAK that we used (0.02%), the interpretation of this experiment is not straightforward, but the data suggest that BAK causes a decrease in CCT that can be partially mitigated by the presence of latanoprost. Notably, the correlation between measurements of total CCT of the same samples made manually and by automated segmentation was high (*r*^2^ was 0.646) (Fig. 2c). Thus, both analysis methodologies led to similar conclusions (*data not shown*). At baseline, there were no correlations between IOP and CCT (*r*^2^ was 0.00374) (Fig. 2d), indicating that within this range of CCT values, CCT likely does not cause a detectable deviation in tonometer readings of IOP.

**Figure 3:**
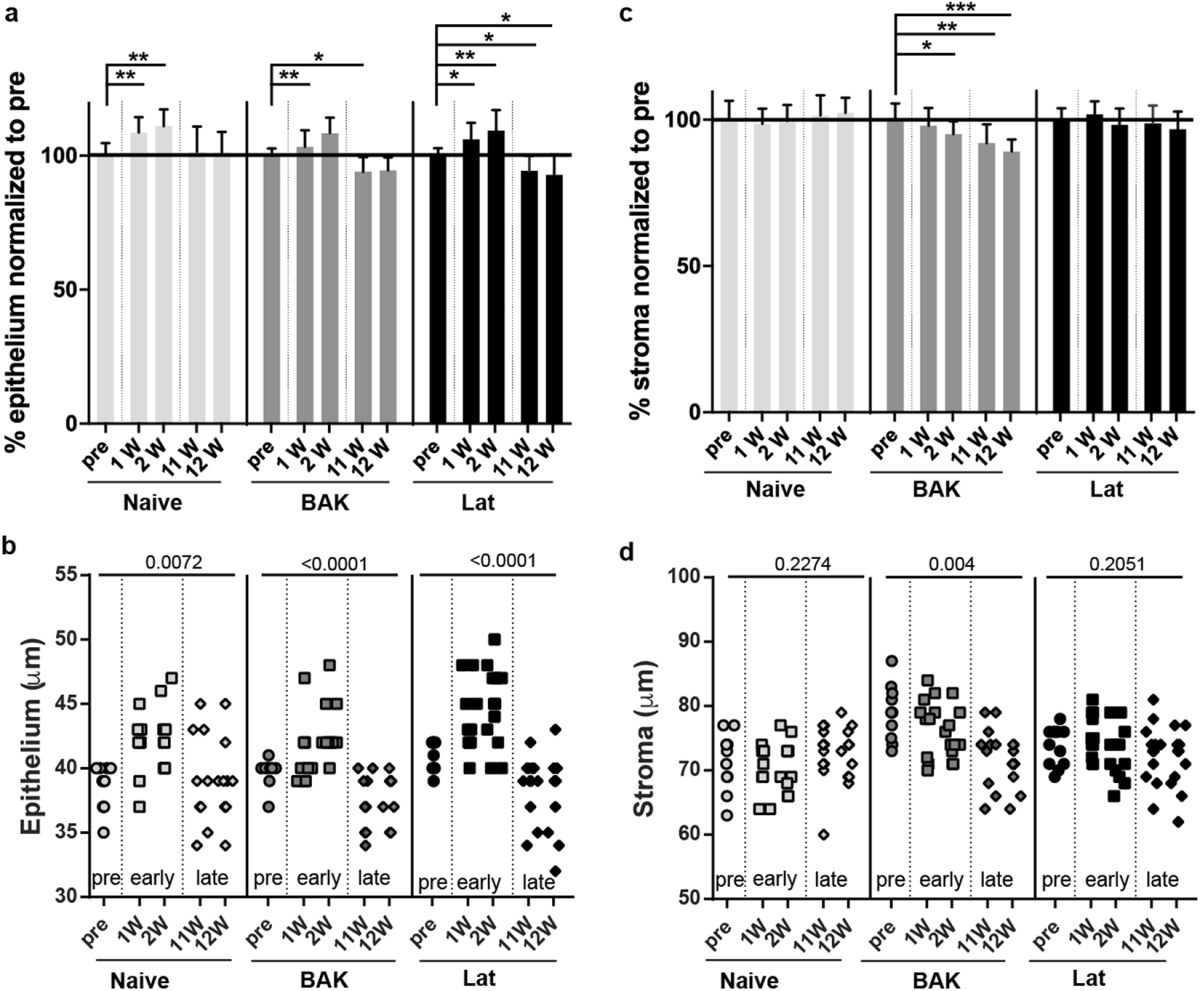
Effects of latanoprost and BAK treatments on the thickness of the epithelium and stromal layers of the central cornea in C57BL/6J mice. (**a**) Mean (±SD) thickness values for epithelium for pre, early (1 week and 2 weeks), and late (11 and 12 weeks) normalized to the mean thickness of the layer determined pre-treatment. (**b**) Scatter plot of all epithelium thickness values with the corresponding *P* value for the repeated measures paired ANOVA analysis indicated. (**c**) Mean (±SD) stroma thickness values for stroma for pre, early (1 week and 2 weeks), and late (11 and 12 weeks) normalized to mean thickness of the layer determined pre-treatment. (**d**) Scatter plot of all stromal thickness values with the corresponding *P* value for the repeated measures paired ANOVA analysis indicated. A repeated measures paired ANOVA analysis with the Dunnett post*-*hoc test was performed and the results of the multiple comparison are shown as: ^*^P < 0.05, ^**^P < 0.01 in (**a**,**c**), respectively. n = 5 mice (Naïve), 5 mice (BAK), and 6 mice (Lat).

To determine if the tested treatments have tissue-specific effects on the cornea, we additionally analyzed the thickness of both the corneal epithelium and the corneal stroma in all samples (Fig. 3). Epithelial thickness, which ranged from 32–50 µm, exhibited a complex pattern of differences, with multiple changes in thickness detected across the treatment groups (Fig. 3a). Notably, sustained treatment led to a significant decrease in epithelial thickness in both the BAK and Lat groups (BAK pre-treatment 39.7 ± 1.06 µm versus BAK week 11 37.3 ± 2.16 µm, *p* = 0.0215; Lat pre-treatment 41.0 ± 1.13 µm versus Lat week 11 38.67 ± 2.27 µm, *p* = 0.018). In contrast, stromal thickness, which ranged from 60–70 µm, showed only a single statistically significant difference across the groups (Fig. 3b), a reduction in thickness for the group treated with BAK long term (BAK pre-treatment 79.0 ± 4.39 µm versus BAK week 11 72.7 ± 5.14 µm, *p* = 0.0053). In summary, these results are reflective of what was observed for total CCT: BAK causes a decrease in CCT that can be partially mitigated in the stroma by the co-presence of latanoprost.

### Sustained treatment with BAK or latanoprost does not change iris pigmentation

**Figure 4:**
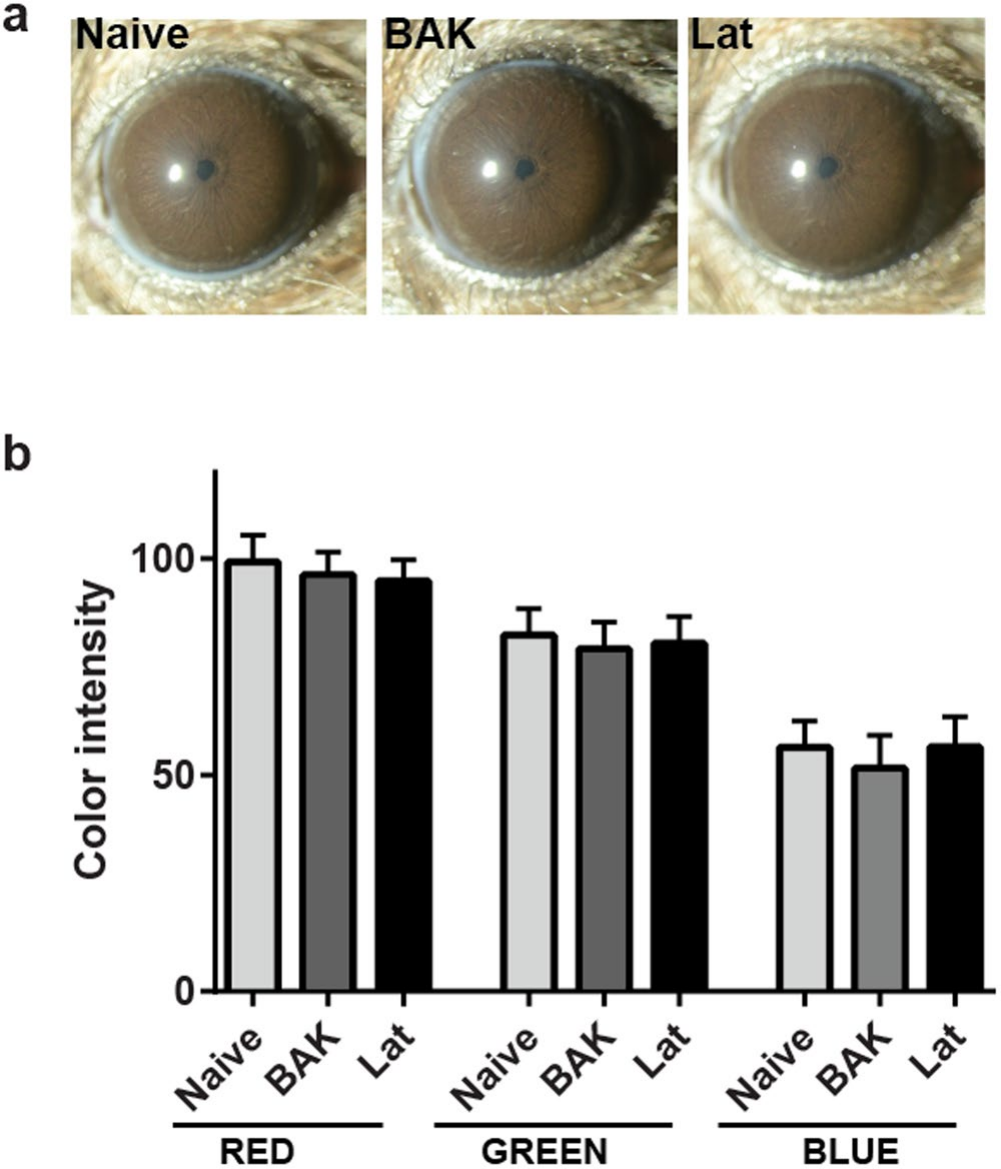
Effects of latanoprost and BAK treatments on iris pigmentation in C57BL/6J mice. (**a**) Representative slit lamp images of eyes for indicated treatment group after 14 weeks of treatment. (**b**) Mean color intensity at 14 weeks, for indicated treatment group. Means ± SD, as determined by ANOVA with Tukey’s multi-comparison post-hoc test. n = 5 mice (Naïve), 5 mice (BAK), and 6 mice (Lat).

Among the known side-effects of latanoprost treatment in humans, darkening of the iris is one of the most common^44^. Latanoprost changes iris pigmentation by stimulating eumelanogenesis^45^, through a mechanism that is thought to involve increased transcription of the tyrosinase gene^46–48^. Although latanoprost is capable of increasing tyrosinase activity in cultured mouse epidermal melanocytes^49^, its effects on mouse iris pigmentation *in vivo* have not been reported. No qualitative differences in iris pigmentation were observed at any time-point after treatment in mice. To qualitatively determine the state of iris pigmentation in the three groups of mice, slit-lamp examination was performed at 14 weeks of treatment. The irides were dark brown, and no overt changes to the iridocorneal angle or lens were present. There were no qualitative (Fig. 4a) or quantitative (Fig. 4b) changes in iris pigmentation between the three groups. Thus, the darkly pigmented iris of C57BL/6 J mice appears to be insensitive to this side-effect of latanoprost. All eyes examined by slit-lamp were characterized by clear corneas, absence of cataracts and normal appearing anterior chambers.

### Long-term treatment with BAK and latanoprost do not cause changes in the expression of matrix metalloprotease or pigment-related genes

**Figure 5:**
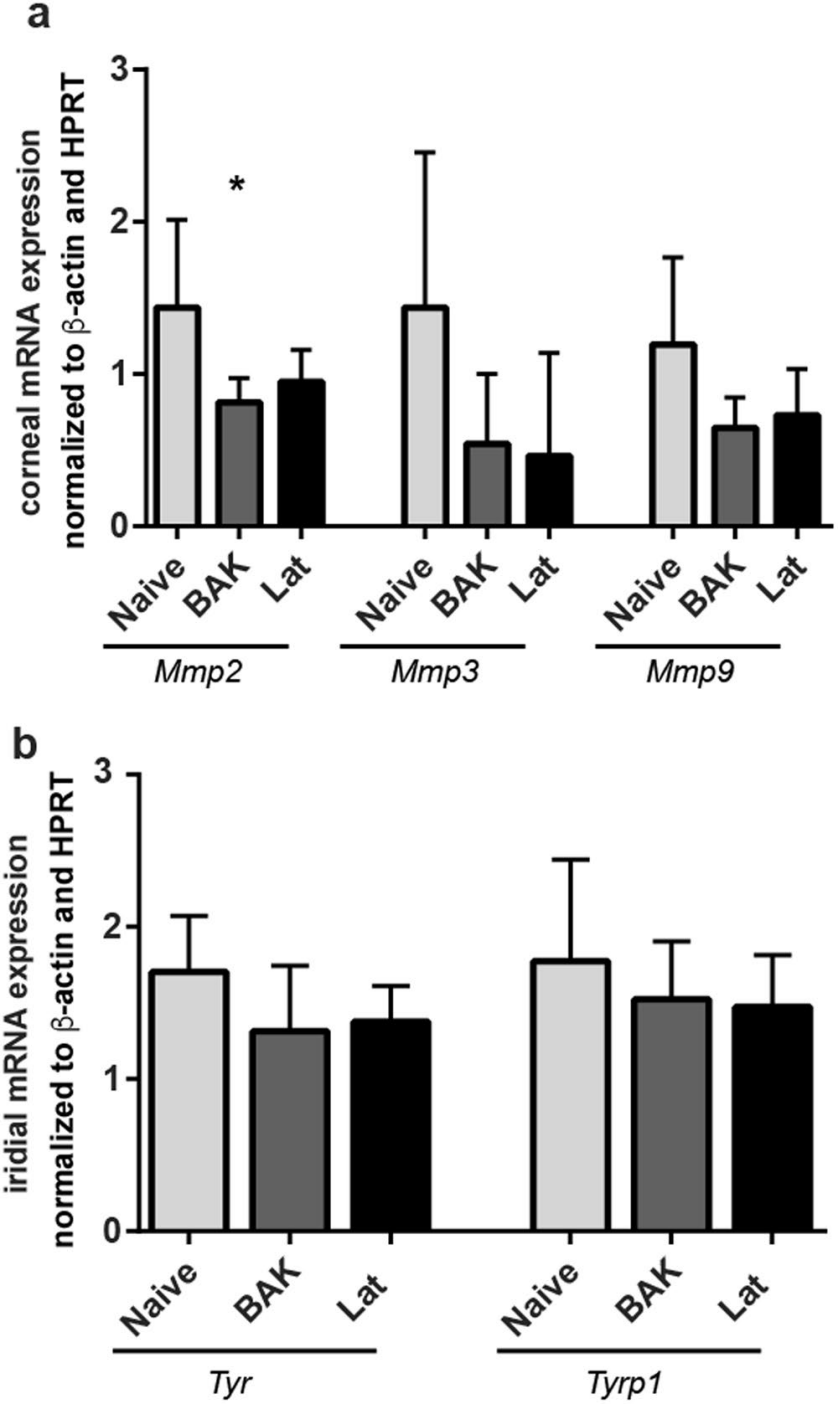
Effects of latanoprost and BAK treatments on corneal and iridial gene expression in C57BL/6J mice. (**a**) Mean levels of *Mmps2*, *Mmps3* and *Mmps9* mRNAs in cornea after 16 weeks of treatment. (**b**) Mean levels of *Tyr* and *Tyrp* mRNAs in iri*s*. Mean ± SD, as determined by ANOVA with Tukey’s multi-comparison post-hoc test. ^*^P < 0.05. n = 5 mice (Naïve), 5 mice (BAK), and 6 mice (Lat).

Several studies have assessed changes in gene transcription following latanoprost treatment^22,50–53^. Thus, at the conclusion of our study, we collected tissues for the analysis of candidate genes in the cornea and iris. Among potential candidates, we chose to focus on *Mmp2*, *Mmp3*, and *Mmp9* in the cornea, and *Tyr* and *Tyrp1* in the iris. RT-PCR revealed only one, significant difference in expression of the *Mmp* genes, a decrease in *Mmp2* mRNA levels in the context of BAK treatment (0.56 fold decrease, *p* = 0.0405) (Fig. 5a). Similarly, RT-PCR-based examination of the iridial expression of *Tyr* and *Tyrp1* in each of the groups revealed no significant differences (Fig. 5b).

## Discussion

The current study shows that in C57BL/6 J mice, latanoprost is an efficient IOP-lowering drug for short-term, but not sustained, treatment. Anatomically, the cornea and iris of treated mice were relatively unaffected by sustained latanoprost treatment. Consistent with findings from other studies^54^, long-term treatment with the preservative present in commercial latanoprost, BAK, leads to corneal thinning. In comparing our findings to those from clinical studies conducted in humans, we noted both similarities and differences, all of which are relevant to ongoing studies of ophthalmic drug development and glaucoma.

A major physiological finding in the current study was that IOP in C57BL/6J mice is initially lowered by latanoprost. In humans, latanoprost treatment has typically been reported to reduce IOP by ~20–35% (starting approximately 2–4 h after the first treatment with the peak effect reached at 8–12 h, and the maximum IOP decrease typically attained 3–5 weeks into the treatment)^55^, with a > 25% improvement often observed in primary open-angle glaucoma patients^56,57^ and <20% improvement more typical in patients with normal tension glaucoma or ocular hypertension^58–60^. Several studies have shown that latanoprost significantly influences uveoscleral outflow^61,62^. Among these there is a relatively small (*n* = 30 participants), but detailed, study of aqueous humor dynamics, which revealed that subjects with ocular hypertension and treated with latanoprost had increased uveoscleral outflow both 1 week and 6 weeks after treatment, and that other components of aqueous humor dynamics were not affected^58^. As expected from previous studies^18,20,21,63^, we observed that with short-term treatment, latanoprost reduced the IOP of standard inbred C57BL/6J mice by 13%. Thus, the initial IOP-lowering response we observed in mice is generally consistent with predictions based on human studies, as well as with other studies showing that mice have an active uveoscleral outflow pathway that influences IOP^21,24^.

The second physiological finding of this study is that after 14–15 weeks of treatment, the mice treated with latanoprost no longer exhibited the improvements observed early in the study. Relatively few studies have specifically attempted to study latanoprost non-responsiveness in humans (Reviewed in^55^), though the phenomenon has been observed repeatedly. For example, Noecker *et al*. found that 28–35% of patients failed to achieve a 15% reduction in IOP after 6 months of daily latanoprost use^64^, and Rossetti *et al*. found that 4.1% of patients failed to achieve a 15% reduction after 1 month of daily treatment with latanoprost^7^. Which factors are predictive of latanoprost responsiveness is not clear, but likely contributors are age^5^, ethnicity^5,65^, and type of glaucoma^66^. Several hypotheses regarding the underpinnings of latanoprost non-responsiveness have been proposed, including possible differences in function or expression of PG receptors, and differences in levels of corneal esterase, an enzyme required for conversion of the latanoprost prodrug to its bioactive form^62,67–72^. Although it was beyond the scope of the current study to attempt to test these hypotheses, our identification of this loss in responsiveness suggests that the C57BL/6J strain of inbred mice would be a tractable resource for a wide variety of mechanistic studies of changes in responsiveness and could promote advances in how latanoprost is used in humans.

One unexpected observation from the IOP study was that sustained latanoprost treatment seemed to not only result in an inability of latanoprost to decrease IOP but may actually elevate IOP (note Lat cohort at late time points in Fig. 1). Though corneas of all mice were clear and apparently undamaged through 14 weeks of treatment, it is possible that Lat treatment at a concentration designed for human eyes was in fact ultimately damaging to mouse eyes with sustained treatment, causing IOP to elevate. Alternatively, the effect could be the consequence of mice becoming hyper-responsive to handling and suffering from a stress-related elevation of IOP. Approximately half of the eyes in this study (across all treatment groups;17/32) had >10% higher IOP measurements at week 14 compared to the base-line (see Supplemental Table 2). At week 15 most of these were closer to baseline, except in the Lat group which tended to remain elevated (see Supplemental Table 2). Regardless, we point out this pattern of IOP elevation as a curiosity that was apparent at both the 14- and 15-weeks time-points.

Our analysis of the influence of latanoprost application on the cornea identified anatomical changes. Although latanoprost is generally well-tolerated, previous studies in a variety of systems revealed that latanoprost formulations that contain the preservative BAK can have detrimental effects on corneal cells. Among these effects are epithelial cytotoxicity^73–75^, an increase in the incidence of ocular surface disease^76–78^, and delays in corneal wound healing^79^. All of these effects are largely dependent on BAK^75,80,73–81^. Although we did not assay most of these parameters related to the surface in the current study, the fact that all of the corneas in all of the treatment groups remained clear indicates that overt surface disease is absent. Nevertheless, based on the findings of Barabino *et al*., who also studied C57BL/6 mice treated with BAK (5 μL of 0.2 mg/mL or 0.02%, 4 times a day for 7 days), we suspect that treatment with solutions containing BAK likely caused sub-clinical damage to the corneal surface in our mice^82^.

A variety of studies in humans have shown that prolonged treatment with latanoprost tends to lead to decreased CCT^41,42,83^. Our current study revealed a similar, though not statistically significant, overall trend toward a decrease in CCT during sustained latanoprost treatment. In our experiment, the thinning appeared to be driven primarily by BAK-associated reductions in thickness of the epithelium and stroma. Our analysis of corneal thickness yielded two unexpected findings. First, measurements of epithelial thickness were unstable; particularly puzzling was a significant increase in thickness in all three groups at the early time points, including in the Naïve cohort. Our current experiments do not distinguish whether this was an effect of age, subtle environmental influences, or epithelial disruption. Biological influences of some of these factors have been partially studied in humans^84^, but much remains unknown. Second, the commercial preparation of latanoprost (which contains both latanoprost and BAK) was associated with less thinning than the BAK solution. Our current experiments do not distinguish whether this was because of a slight difference between our BAK formulation and the proprietary commercial solution, or a biological feature of latanoprost that can mitigate thinning associated with BAK. Several previous studies also found that BAK on its own is more detrimental than BAK with a PG, though those studies primarily assessed different phenotypes^75,79,85^.

The molecular consequences of latanoprost application that influence ocular anatomy and physiology remain incompletely understood, but in the cases of the effects on both IOP^86–88^ and the cornea^51^, they likely involve activation of matrix metalloproteinases, which have independently been implicated in glaucoma in multiple ways^89,90^. With respect to corneal gene transcription, we tested transcript levels of *Mmp3* and *Mmp9*, both of which were previously found to be increased in the tears of PG-treated patients^51^ as well as in various types of corneal wounds^91,92^. In addition, in the cornea and conjunctiva of ddY mice, levels of *Mmp9*, but not *Mmp1*, were increased after two months of treatment with latanoprost^22^. Thus, we hypothesized that BAK or latanoprost might elevate levels of *Mmp2*, *Mmp3*, and/or *Mmp9* transcripts, an outcome that might have accounted for the corneal thinning in this context. Our finding that BAK is associated with only a single statistically significant difference, a modest reduction in levels of the *Mmp2* transcript, is inconsistent with a finding reported by Sharma *et al*. in rats. This group studied the influence of BAK on wounded rat corneas and found no statistical difference in *Mmp2* transcript levels^91^. Furthermore, our results are inconsistent with the observation that levels of the *Mmp9* transcript increase in ddY mice after two months of treatment with latanoprost^22^. We presume that methodological differences in the studies underlie the differing outcomes; additional experiments would be necessary to test this possibility. In summary, our experiments did not identify any increase in levels of transcription of these candidate *Mmps* that provide insight into why sustained BAK treatment leads to corneal thinning in mice.

The final ocular phenotype we examined in the current study was iris pigmentation. Notably, this typically increases in humans following sustained latanoprost use^2^, for example 12, 23 and 11% of patients from USA, United Kingdom and Scandinavia after one year of treatment^93^, but was unaltered by our treatment regime in C57BL/6J mice. As with other actions of latanoprost, the mechanism leading to iris darkening is incompletely understood. However this side-effect is common to all of the IOP-lowering PG analogs^9–11^, occurs in the absence of BAK^94^, and tends to be more pronounced in eyes with certain iris colors (green-brown, yellow-brown)^95^. Interestingly, studies from Japan have shown that even in eyes with brown irises, darkening is present (up to 60%)^44,96^, which is conflicting with what was observed in studies from Europe and USA. Latanoprost was previously found to stimulate both the transcription of tyrosinase, the rate-limiting catalyst of melanin synthesis in melanosomes, in iridial melanocytes of cynomologus monkeys, cultivated iridial melanocytes and iridial tissue from humans^47^. Increased activity of tyrosinase was detected in cultured human iridial melanocytes and human cell lines^47^. One possible, though speculative, explanation for the species-dependent difference that we observed in mice relates to the abundance of iris fibroblasts. These cells have been proposed to be important in mediating the pigment stimulating effect of latanoprost in melanocytes^97^, and are much more abundant in humans than in mice^31^.

Although this study uncovered previously unknown aspects of sustained latanoprost treatment in mice, it has caveats that warrant mention. First, our experimental design included completely random, pre-treatment division of mice into 3 study groups. Despite the fact that all the mice used were inbred and obtained from The Jackson Laboratory as part of a single shipment, the mean CCT values for the study groups by chance differed from the outset (compare the pre-treatment time points of each group in Fig. 2b). This peculiarity highlights the importance of this challenge, which is inherent to the random design approach, which we had used in an effort to promote robustness. Because of this result, our data are presented with and without normalization, in case the differing baseline values resulted in biases that skewed analyses. Second, our protocol for measuring IOP specified a defined window of time: 2–4 hours post-treatment. Our study did not address whether the observed changes in responsiveness to latanoprost might be a consequence of differing kinetic effects that might have been more apparent at different times post-treatment^18,20,63^, or other circadian periods. Third, an important methodological distinction complicates the direct comparison of our results to humans: both groups of time points studied (after 1 and 2 versus 14 and 15 weeks of treatment) could be considered “early” in clinical practice, i.e., the observed loss of responsiveness might be considered an absence of responsiveness in humans. Fourth, we did not include a cohort treated with a commercial preservative-free formulation of latanoprost as a control, thus our study does not address the consequences of treatment with latanoprost alone. Fifth, our study utilized the Lat formulation used in humans and also used by other researchers in mice^21,27,63^. Sixth, our experimental design centered on average group responses, not the response of individual mice. This design was selected because the mice were all genetically identical, sex-matched and environment matched, and IOP measurements in mice are influenced by many factors that are difficult to standardize. Thus, our design did not address the possibility of individual non-responders, an issue that would be better addressed using outbred rather than inbred mice.

Our results also have three additional specific implications that will be of particular interest to scientists using mice to study glaucoma^98–100^. The first is that long-term treatment with latanoprost may not be sufficient to ameliorate glaucoma in mouse models characterized by slow neurodegeneration. Second, in mice, IOP measurements involving rebound tonometry do not correlate with CCT. Among these genetically identical mice subjected to differing treatments, changes in IOP were not predictive of changes in CCT or vice versa. Third, for any mouse study in which latanoprost-treatment might be a co-variate, its main effect seems to be on IOP.

In sum, this study was designed to identify the ocular consequences of latanoprost treatment in mice on a schedule that mimics the application of drops in human glaucoma patients, and over a prolonged period of time. The results suggest that latanoprost is an efficient IOP-lowering drug for short-term, but not sustained, treatment, in this strain of mice, and that its sustained use might lead to mild corneal thinning (statistically insignificant trend) without affecting iris pigmentation in this strain of mice with dark brown irides. Our current results do not distinguish whether this loss of responsiveness is a feature of all laboratory mice, or might be dependent on the genetic background, a worthwhile question to pursue in ongoing work.

## Electronic supplementary material

Supplementary information
