# Supplementary material for: Effects of sustained daily latanoprost application on anterior chamber anatomy and physiology in mice
Source: Sci Rep. 2018 Aug 30;8:13088. doi: 10.1038/s41598-018-31280-1 (PMC6117323; doi:10.1038/s41598-018-31280-1)
Supplement: Supplementary file 1 — Supplementary information [file 41598_2018_31280_MOESM1_ESM.docx]

**Supplementary information**

**Effects of sustained daily latanoprost application on anterior chamber anatomy and physiology in mice**

Laura M. Dutca^1,2^, Danielle Rudd^1^, Victor Robles^3^, Anat Galor^4^, Mona K. Garvin^1,3^; Michael G. Anderson^1,2,5.^

^1^Center for Prevention and Treatment of Visual Loss Iowa City Veterans Administration Medical Center, Iowa City, IA; ^2^Department of Ophthalmology and Visual Science, University of Iowa, Iowa City, IA; ^3^Electrical and Computer Engineering, University of Iowa, Iowa City, IA; ^4^Miami Veterans Administration Medical Center and Bascom Palmer Institute, University of Miami, Miami, FL; ^5^Molecular Physiology and Biophysics, University of Iowa, Iowa City, IA.

***Corresponding author:**

Dr. Michael G. Anderson, Department of Molecular Physiology and Biophysics, 3123 Medical Education and Research Facility, 375 Newton Road, Iowa City, IA 52242, 1-319-355-7839 (telephone), 1-319-335-7330 (FAX), [michael-g-anderson@uiowa.edu](mailto:michael-g-anderson@uiowa.edu)


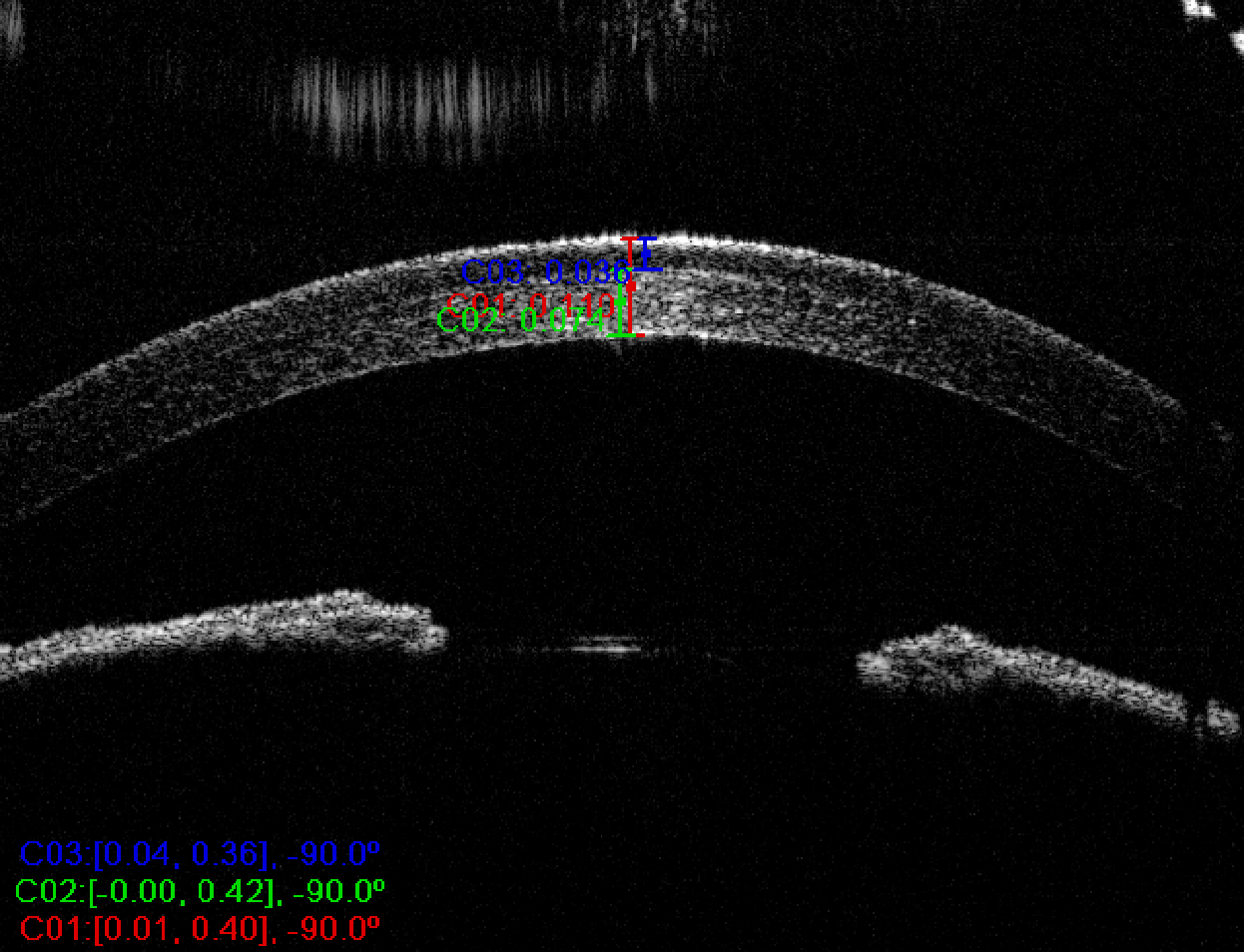


**Supplemental Figure S1**. An optical coherence tomography (OCT) image of the cornea with the placement of the calipers for measuring the total central corneal thickness (red), and the thicknesses of the epithelium (blue) and the stroma (green).

**
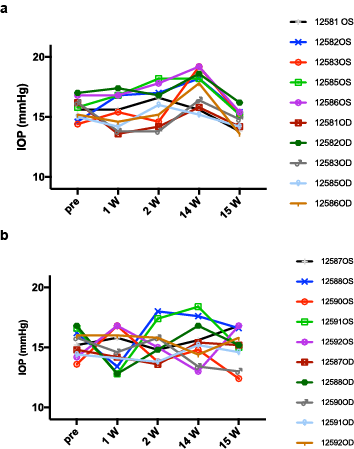
**

**Supplemental Figure S2**. Intraocular pressure measurements over time, with each line corresponding to one eye. The values for the Naïve group are presented in a, while the ones for the BAK treated group are in b.

**Supplemental Table S1**. Primer sequences used for quantitative RT-PCR

| Target mRNA | Primer sequences (5’-3’) | Product size (bp) |
| --- | --- | --- |
| Beta-actin | CATCCGTAAAGACCTCTATGCCAAC  ATGGAGCCACCGATCCACA | 171 |
| HPRT | GTTGGGCTTACCTCACTGCT  ATCGCTAATCACGACGCTGG | 130 |
| Mmp2 | CAGTGATGGCTTCCTCTGGT  GTAAACAAGGCTTCATGGGGG | 87 |
| Mmp3 | CCAGGGATTAATGGAGATGC  CAAGTTCATGAGCAGCAACC | 97 |
| Mmp9 | GCGTCATTCGCGTGGATAAG  CCTGGTTCACCTCATGGTCC | 156 |
| Tyr | ACACACTGGAAGGATTTGCC  AAATGGGATCGTTGGCCGAT | 132 |
| Tyrp1 | CCGCTTTTCTCACATGGCAC  TCGCAGACGTTTTTCCCAGT | 124 |

**Supplemental Table 2**. Intraocular pressure values in mmHg.

*the IOPs were excluded based on the inclusion criteria mentioned in Materials and Methods
